# Moving from a taxonomic to a functional perspective in global microbiome analysis requires optimizing multiplexing ratios

**DOI:** 10.1128/msystems.00144-26

**Published:** 2026-05-29

**Authors:** Kinga Zielińska, Kateryna Pantiukh, Elin Org, Paweł P. Łabaj, Tomasz Kosciolek

**Affiliations:** 1 Małopolska Centre of Biotechnology, Jagiellonian University 37799, Kraków, Poland; 2 Estonian Genome Centre, Institute of Genomics, University of Tartu 37546, Tartu, Estonia; 3 Sano Centre for Computational Medicine 604699, Kraków, Poland; Qingdao University, Qingdao, Shandong, China; Harvard University, Allston, Massachusetts, USA; The University of Hong Kong, Hong Kong, Hong Kong

**Keywords:** metagenomics, microbiome, short-read sequencing

## Abstract

Next-generation sequencing has revolutionized microbiome research, yet the transition from taxonomic to functional profiling remains a major technical challenge. While marker gene sequencing provides a widely accessible ecological view, it often lacks the resolution for actionable insights. This perspective argues that shifting to whole metagenomic sequencing is essential for mapping functional potential, such as antimicrobial resistance, and metabolic pathways. However, we identify a critical bottleneck: excessive multiplexing. High multiplexing ratios reduce the number of unique molecules per sample, leading to high duplication rates and the stochastic dropout of low-abundance genes. We demonstrate that functional profiles are far more sensitive to these library complexity issues than taxonomic ones. We recommend prioritizing total sequencing depth and reducing multiplexing to ensure sufficient unique coverage. Additionally, adopting long-read or hybrid architectures is vital for providing the genomic context necessary for strain-level resolution. These optimizations are prerequisites for robust global microbiome synthesis and translational science.

## PERSPECTIVE

## MOVING FROM TAXONOMIC TO FUNCTIONAL PROFILING

The introduction of next-generation sequencing (NGS), also known as massive parallel sequencing, into microbiome characterization has revolutionized our understanding of microbial communities. Initially, 16S rRNA gene sequencing (and, to a lesser extent, other marker genes such as 18S and ITS sequencing [1]), primarily using short-read technologies, offers a widely accessible but limited view. It provides relatively low taxonomic resolution because it targets only selected hypervariable regions of a single marker gene, limiting the ability to discriminate between closely related taxa at the species and strain level (2). Despite this limitation, 16S-based surveys have enabled an ecological perspective on the microbiome, improving our understanding of taxonomic structure, diversity, and global patterns of microbiome variation (3–7). However, while this approach has helped identify major drivers of microbial diversity and test ecological hypotheses in microbial communities, it often falls short of yielding actionable conclusions (8). The advent of long-read sequencing has improved 16S-based taxonomic resolution by covering the gene at full length, but distinguishing closely related species or genera, and especially strains, remains challenging (9). Moreover, marker gene sequencing does not directly support functional characterization beyond genome-level prediction (10).

The shift toward whole metagenomic sequencing (WMS) represents a major leap forward, albeit at the cost of substantially deeper sequencing. WMS, particularly when paired with long-read technologies, can provide higher taxonomic resolution and, when coverage is sufficient, enable the reconstruction of metagenome-assembled genomes and genomic context. Crucially, WMS extends beyond mere taxonomic profiling by enabling the investigation of functional potential, including the detection of specific genes and the identification of functionally relevant features such as antimicrobial resistance (AMR) determinants (11, 12). Applying these capabilities, we contributed to the Metagenomics and Metadesign of Subway and Urban Biomes (MetaSUB) consortium’s development of a high-resolution global metagenomic atlas. Our participation in this initiative focused on leveraging WMS to map urban biomes at scale, thereby translating raw genomic data into actionable public health insights and a culture-independent map of urban AMR. The atlas, containing 4,728 metagenomic samples from mass-transit systems across 60 cities collected over 3 years, represents the first systematic, worldwide catalog of the urban microbial ecosystem (11, 13, 14). Developing this concept for gut microbiome characterization within the q2-predict-dysbiosis framework, we demonstrated that functional potential—in contrast to taxonomic composition—exhibits a more conserved and universal prevalence across global populations (15). While defining a “core” set of globally prevalent taxa in the gut remains nearly impossible due to geographic and individual variability, we identified a vast repertoire of metabolic pathways shared across healthy cohorts worldwide. These findings support the view that metagenomic function provides a more robust and stable lens for global microbiome comparison than phylogeny alone. This is consistent with earlier observations by Turnbaugh et al., who demonstrated in a large twin cohort that functional gene categories are far more conserved between individuals than species-level composition, suggesting that functional redundancy buffers community-level metabolic output against taxonomic variation (16).

## UNDERSTANDING EXPOSOME AND TOWARD OneHealth/PERSONALIZED MEDICINE

Functional profiling is also central to understanding the human exposome and to developing OneHealth solutions that integrate microbiomes across environments. Early steps have been enabled by large consortia, such as International MetaSUB and the Earth Microbiome Project, which provide essential context by cataloging microbial diversity across the planet and helping to distinguish between natural environmental baselines and anthropogenic shifts in built environments (3). In parallel, ongoing work aims to map microbiomes (mostly the gut microbiome) across diverse human cohorts worldwide (5–7, 17–25). Integrating information across microbiomes living around us, on us, and inside us will require a better understanding of how these communities interact and co-vary over time. Realizing these goals depends critically on the reliability of functional profiling across studies and environments. High multiplexing disproportionately causes dropout of low-abundance functional genes, including horizontally transferred elements and lineage-specific AMR determinants that are of greatest epidemiological relevance. Missing these signals systematically across large cohorts does not merely introduce noise but also creates reproducible blind spots in surveillance that could mask the emergence of resistance hotspots or the cross-environment transfer of resistance determinants before clinical consequences become apparent. Ensuring adequate library complexity is therefore not only a technical concern but also a prerequisite for the population-level functional inferences that exposome monitoring and global AMR surveillance depend upon. Such advances could support microbial “digital twins” of global, local, and personal exposomes and contribute to preventive and personalized medicine.

To make it happen we need technologies able to generate high quality, deeply sequenced data and methodological advances in comparable functional inference across studies and platforms.

## CHALLENGES IN FUNCTIONAL ANALYSES STEM FROM PRE-SEQUENCING PROTOCOLS

Moving from taxonomic to functional potential profiling introduces new complexities, particularly concerning data quality. For taxonomic analysis, the primary requirement is that reads can be attributed to organisms (or their genomic regions) in a way that reflects abundance. For accurate functional potential assessment, an additional critical requirement emerges: reasonably uniform unique coverage across the region(s) of interest (e.g., genes, operons, and genome). This is strongly influenced by wet-lab procedures. Experimental design and execution choices that have minimal impact on the stability and reproducibility of taxonomy-based analyzes can substantially compromise functional profiles.

Accordingly, the influence of wet-lab procedures on NGS results is a major focus of large international efforts such as US FDA MAQC/SEQC and ABRF (26, 27). For instance, a recent ABRF consortium manuscript, although focused on RNA-Seq, reported a strong correlation between low amounts of starting material and high duplication rates, with downstream effects on concordance between sequencing technologies (28). Related challenges were highlighted more than a decade ago, when standard library preparation methods were reported to cause a loss of rare transcripts (29). Importantly, analogous limitations have been documented in metagenomic contexts: Hillmann et al. demonstrated that shallow shotgun sequencing imposes a fundamental ceiling on functional analysis that does not apply equally to taxonomic profiling, as low sequencing depth precludes *de novo* assembly of novel genes and genomic regions entirely (30).

To minimize costs, metagenomic studies commonly use large sequencing instruments with high multiplexing. While efficient, sequencing many samples at once often reduces per-sample input material and library complexity. Low input can increase duplication rates and degrade the evenness of coverage across microbial genomes, leading to a systematic dropout of low-to-moderate abundance genes. Although it was reported recently that low-prevalence functions are disproportionately lost at shallow sequencing depths (31), we understand now that the sequencing depth is only an indirect cause here. Our recent analysis of matched human metagenomic samples, sequenced twice with different protocols, highlights a critical pitfall in global microbiome comparisons: the trade-off between multiplexing and functional resolution. While taxonomic assignments at the species and genus level can be relatively resilient because they are supported by multiple genomic regions and redundant marker sequences, functional profiling depends on unique hits to specific genes and domains. Importantly, this resilience does not extend to strain-level taxonomy: tools such as inStrain and StrainPhlAn require substantial uniform per-genome coverage to resolve variants reliably and are therefore subject to the same library complexity constraints as functional annotation, further underscoring the need for adequate unique coverage in studies aiming for strain-resolved or functional interpretation. Crucially, this is not simply a matter of sequencing depth: a library prepared under high multiplexing conditions has inherently reduced molecular complexity, and no amount of additional sequencing from that same pool can recover molecules that were never captured. This makes high multiplexing an upstream, irreversible constraint on functional resolution, distinct from, and not correctable by, increasing read depth alone (32). Our companion analysis of 1,351 matched sample pairs provides direct empirical support: after rarefaction to identical read depths, samples processed with lower multiplexing retained 5–10 times more unique k-mers than their high-multiplexing counterparts (Fig. 5D in reference 32), and functional distances between protocols persisted at approximately double the intra-platform baseline despite matched depth (Fig. 5H in reference 32). These results confirm that the problem is one of library complexity—determined upstream at the multiplexing stage—rather than read depth *per se*.

## ESTABLISHING MICROBIAL GENE CATALOGS

At present, multiple efforts, spanning a variety of biomes, attempt to catalog and characterize the genetic diversity of microbiome-derived genes (33–37). Notably, the Unified Human Gastrointestinal Protein (UHGP) catalog and the more recent Gut Microbiome Reference indicate that taxonomic and functional diversity within the global human gut microbiome remains underexploited: we have not yet saturated the number of species or gene/protein clusters (33, 34). How much of this gap reflects undersampling of populations vs technical artifacts of experimental design and sequencing strategy remains an open question.

## COMPUTATIONAL CHALLENGES GO HAND-IN-HAND WITH EXPERIMENTAL CONSIDERATIONS

The transition from descriptive to functional interpretation of the human microbiome is fundamentally driven by advances in bioinformatics methodology. To date, functional profiling in metagenomics has been dominated by homology-based approaches, including widely used pipelines such as HUMAnN2/3 (38) and eggNOG (39) or other specialized mapping-based approaches like mi-faser (40). These methods have proven practical and scalable, but their limitations are intrinsic to their design: functional inference is constrained by sequence similarity to existing reference databases, and therefore by the completeness and bias of current annotations.

Despite the human gut microbiome being the most extensively studied microbial ecosystem to date, functional annotation remains strikingly incomplete. Large-scale efforts such as the UHGP catalog indicate that only ~60% of currently known and cataloged gut microbial genes can be assigned a function. This figure reflects annotation limits for already discovered genes; it does not account for the substantial fraction of genes that may remain undetected due to methodological constraints in sequencing, assembly, and coverage. Our recent work (32) demonstrates that even in deeply sequenced cohorts, a non-trivial proportion of genes that may be present in samples remains systematically missed, further widening the gap between biological reality and functional interpretation. Critically, these two gaps—technical and computational—are not independent. High multiplexing causes stochastic dropout that disproportionately affects rare, low-abundance, and lineage-specific genes: the same genes that are most poorly represented or entirely absent from existing reference databases, which are themselves biased toward the abundant functions detected most frequently across studies. This creates a compounding effect: low-complexity libraries reduce the probability of capturing rare genes in sequencing, and if those genes are captured at reduced coverage, incomplete reference databases reduce the probability of annotating them correctly. Computational advances—however sophisticated—struggle to annotate molecules that were never sequenced. This reinforces the urgency of the wet-lab recommendations we propose below, and explains why improving library complexity is a prerequisite for, rather than an alternative to, methodological progress in functional annotation.

Several emerging approaches aim to alleviate these limitations. Deep learning-based methods for function prediction, such as deepFRI and related work, attempt to move beyond strict homology by learning continuous representations of function directly from sequence and 3D structure (40, 41). Parallel progress has been made in enzyme and EC number annotation, where the functional label space is more constrained and better defined, as exemplified by methods such as PARSE and CLEAN (42, 43). While these approaches differ in scope and maturity, collectively, they signal a clear methodological shift away from purely homology-driven inference. In practice, deep learning-based methods such as deepFRI are most valuable where homology-based pipelines are weakest: for annotating novel, lineage-specific, or poorly characterized genes that lack close homologs in reference databases, precisely the genes most likely to be missed or misannotated by tools such as HUMAnN or eggNOG.

Looking forward, an integrated sequence–structure–function view of the microbiome is becoming technically feasible. High-quality structure prediction at scale, enabled by AlphaFold and related models, has largely transformed our ability to reason about protein structure (44, 45). In parallel, representation learning approaches to protein function are beginning to provide quantitative, model-based alternatives to discrete ontology assignment. Realizing the full potential of this convergence will depend critically on the availability of high-quality, deeply sequenced, and methodologically consistent metagenomic data sets. Without such data, advances in modeling will remain bottlenecked by incomplete, biased, or irreproducible functional signals.

In this context, improving experimental design, sequencing depth, and cross-platform consistency is not merely a technical concern: it is central to the scientific goal of moving from descriptive to mechanistic and translational microbiome science.

## RECOMMENDATIONS FOR FUNCTIONAL PROFILING

To fully unlock the potential of functional metagenomics and ensure robust, reproducible results, we propose a strategic shift in how we approach the “wet lab-to-bioinformatics” pipeline.

- Prioritize coverage depth over sample volume**.** We recommend deliberately reducing multiplexing levels, potentially using fewer samples per sequencing lane, in order to achieve sufficient minimal per-sample coverage depth for reliable gene and pathway detection. This technical pivot ensures a more even distribution of reads across the metagenome, significantly reducing the stochastic “dropouts” of rare but functionally critical enzymes and metabolic genes.
  Establishing quantitative guidelines for multiplexing will require systematic titration experiments across community types and sequencing configurations. Our companion analysis of 1,351 matched sample pairs (32) provides an empirical foundation for such efforts: even after rarefaction to identical read depths, samples prepared with lower multiplexing retained 5–10 times more unique k-mers and detected significantly more enzymes, with 241 EC numbers showing systematic platform-dependent bias. These data demonstrate that the relationship between multiplexing level and functional recovery is quantifiable, and we anticipate that future work integrating variables such as total sequencing output, community diversity, and target functional complexity could yield practical frameworks for optimizing multiplexing ratios in study design.
  Importantly, the cost implications of reducing multiplexing are real but should be evaluated in terms of information per valid functional data point rather than cost per sample: data generated at high multiplexing may have limited utility for functional questions regardless of the investment made. A practical path forward for large cohort studies is a tiered design—high multiplexing for full-cohort taxonomic profiling, with a well-powered lower-multiplexing subset reserved for functional analyses—which optimizes information yield across both objectives without requiring that every sample be sequenced at maximum depth. Where taxonomic profiling is the primary objective, higher multiplexing ratios remain a valid and cost-effective strategy, as our companion analysis (32) demonstrates robust taxonomic concordance (>92% species overlap) even under high-multiplexing conditions. The rapidly declining cost of sequencing per gigabase further suggests that this trade-off will become increasingly favorable for functional studies in the near term.
- Transition to long-read architectures**.** The field must increasingly embrace long-read sequencing technologies. Unlike fragmented short reads, long reads can span entire genes, operons, and complex functional domains, providing a superior scaffold for functional interpretability. Crucially, this continuity enables strain-level resolution, allowing us to map specific functional potentials to the precise organisms responsible for them: a granularity that is often lost in the “taxonomic noise” of short-read assemblies. As an alternative to relying solely on the long-read technology, we recommend a hybrid sequencing approach. Such a design combines deep short reads with targeted long-read data and may offer a cost-effective compromise, improving functional resolution without prohibitive expense.
  We note, however, that long-read functional metagenomics remains a specialist technique rather than a turnkey solution. Although recent rapid advancements have significantly improved per-base error rates, bringing them close to those of short-read technologies, these errors can still complicate precise functional assignment at the nucleotide level. They can be particularly problematic for AMR gene variant identification, where even a single SNP can determine resistance profiles. It is worth noting, however, that some long-read sequencing technologies have recently been approved for diagnostic use. Together with emerging innovations in the field, this suggests that long-read functional metagenomics may soon become a turnkey solution. The hybrid approach, while the most pragmatic path forward, requires both short-read polishing and long-read assembly infrastructure that many laboratories currently lack. We therefore present these as directional recommendations for the field rather than immediately actionable prescriptions for every research group and anticipate that continued improvements in long-read accuracy and the development of dedicated metagenomic long-read pipelines will progressively lower these barriers.
  Additionally, complementary experimental strategies, such as targeted enrichment of specific functional gene classes (e.g., antimicrobial resistance gene panels), can provide focused functional resolution where comprehensive whole-metagenome coverage is not achievable, although these approaches are inherently limited to predefined gene targets and do not substitute for unbiased metagenomic profiling.

**Fig 1:**
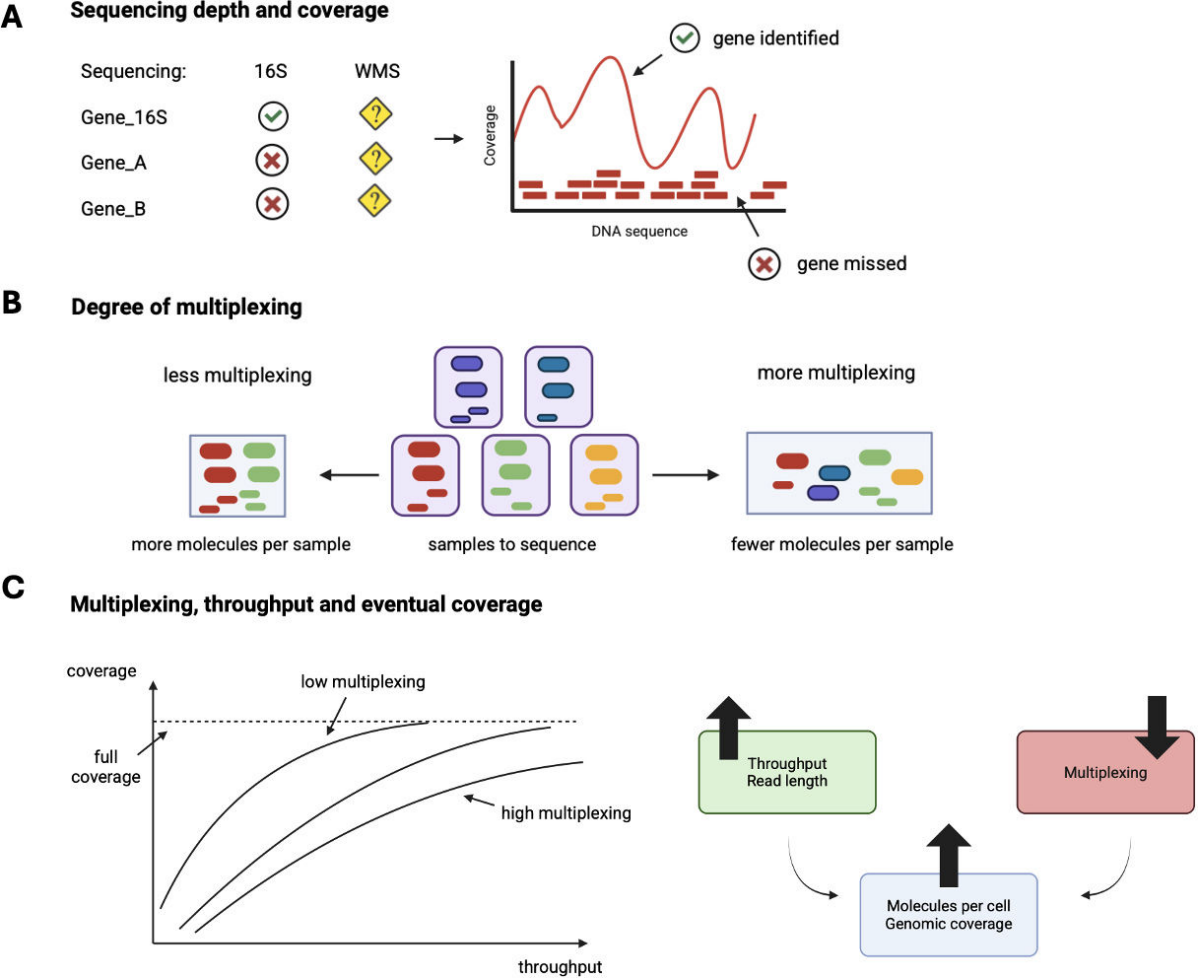
Interplay between sequencing depth, multiplexing, and coverage in metagenomic profiling. (**A**) Comparative analysis of sequencing coverage across platforms, highlighting the limitations in identifying specific gene targets (e.g., 16S rRNA vs whole metagenomic sequencing), where low coverage may result in “missed” annotations (dropouts). (**B**) Impact of multiplexing depth on molecular recovery. At high degrees of multiplexing, reduced per-sample input means that rare molecules are frequently absent from the starting material by stochastic chance. Sequences present at low copy number may not be sampled from the limited input pool. Subsequent PCR amplification overrepresents whichever templates were captured, producing high duplication rates and a library whose apparent complexity masks the systematic absence of rare sequences that were never present in the initial input. (**C**) The relationship between throughput and eventual genomic coverage; the saturation curves illustrate how higher multiplexing requires significantly greater total throughput to achieve the coverage depth necessary for accurate molecule identification and comprehensive genomic reconstruction. These curves are schematic representations of relationships empirically demonstrated in matched-sample data in reference 31; see Fig. 5D and H therein for the underlying data.

By addressing these critical wet-lab and sequencing strategy considerations, we can overcome current challenges and fully realize the transformative potential of high-resolution, functional metagenomics (Fig. 1). This is crucial not only for achieving more accurate and robust insights within individual studies but also for harmonizing data across diverse cohorts and data sets, enabling powerful meta-analyses that are currently hindered by inconsistencies arising from varied sequencing protocols. Ultimately, these advancements will move the field toward a deeper and more comprehensive understanding of microbial activity across diverse environments.

## Supplementary Material

Reviewer comments

## SUPPLEMENTAL MATERIAL

OPEN PEER REVIEW reviewer-comments.pdf An accounting of the reviewer comments and feedback.

ASM does not own the copyrights to Supplemental Material that may be linked to, or accessed through, an article. The authors have granted ASM a non-exclusive, world-wide license to publish the Supplemental Material files. Please contact the corresponding author directly for reuse.
