# Supplementary material for: Moving from a taxonomic to a functional perspective in global microbiome analysis requires optimizing multiplexing ratios
Source: mSystems. 2026 May 29;11(6):e00144-26. doi: 10.1128/msystems.00144-26 (PMC13289057; doi:10.1128/msystems.00144-26)
Supplement: Reviewer comments [file reviewer-comments.pdf]

AMERICAN SOCIETY FOR MICROBIOLOGY | mSystems®

Moving from taxonomic to functional perspective in global microbiome analysis requires optimizing multiplexing ratios

Kinga Zielińska, Kateryna Pantiukh, Elin Org, Pawel Labaj, and Tomasz Kosciolek

Corresponding Author(s): Tomasz Kosciolek, Sano Centre for Computational Personalised Medicine

Review Timeline:

Submission Date:	February 5, 2026
Editorial Decision:	March 30, 2026
Revision Received:	April 22, 2026
Accepted:	May 8, 2026

Editor: Xiaoquan Su

Reviewer(s): Disclosure of reviewer identity is with reference to reviewer comments included in decision letter(s). The following individuals involved in review of your submission have agreed to reveal their identity: Ya Wang (Reviewer #1); Shi Huang (Reviewer #2)

Transaction Report:

(Note: With the exception of the correction of typographical or spelling errors that could be a source of ambiguity, letters and reports are not edited. The original formatting of letters and referee reports may not be reflected in this compilation.)

DOI: https://doi.org/10.1128/msystems.00144-26

Re: mSystems00144-26 (Moving from taxonomic to functional perspective in global microbiome analysis requires optimizing multiplexing ratios)

Dear Dr. Tomasz Kosciolek:

Thank you for the privilege of reviewing your work. Below you will find my comments, instructions from the mSystems editorial office, and the reviewer comments.

Please return the manuscript within 60 days; if you cannot complete the modification within this time period, please contact me. If you do not wish to modify the manuscript and prefer to submit it to another journal, notify me immediately so that the manuscript may be formally withdrawn from consideration by mSystems.

Revision Guidelines
To submit your modified manuscript, log into the submission site at https://msystems.msubmit.net/cgi-bin/main.plex. Go to Author Tasks and click the appropriate manuscript title to begin. The information you entered when you first submitted the paper will be displayed; update this as necessary. Note the following requirements:

• Upload point-by-point responses to the issues raised by the reviewers in a file named "Response to Reviewers," NOT in your cover letter.
• Upload a compare copy of the manuscript (without figures) as a "Marked-Up Manuscript" file.
• Upload a clean .DOC/.DOCX version of the revised manuscript and remove the previous version.
• Each figure must be uploaded as a separate, editable, high-resolution file (TIFF or EPS preferred), and any multipanel figures must be assembled into one file.
• Any supplemental material intended for posting by ASM should be uploaded with their legends separate from the main manuscript. You can combine all supplemental material into one file (preferred) or split it into a maximum of 10 files with all associated legends included.

For complete guidelines on revision requirements, see our Submission and Review Process webpage. Submission of a paper that does not conform to guidelines may delay acceptance of your manuscript.

Data availability: ASM policy requires that data be available to the public upon online posting of the article, so please verify all links to sequence records, if present, and make sure that each number retrieves the full record of the data. If a new accession number is not linked or a link is broken, provide mSystems production staff with the correct URL for the record. If the accession numbers for new data are not publicly accessible before the expected online posting of the article, publication may be delayed; please contact production staff (mSystems@asmusa.org) immediately with the expected release date.

Publication Fees: For information on publication fees and which article types are subject to charges, visit our website. If your manuscript is accepted for publication and any fees apply, you will be contacted separately about payment during the production process; please follow the instructions in that e-mail. Arrangements for payment must be made before your article is published.

ASM Membership: Corresponding authors may join or renew ASM membership to obtain discounts on publication fees. Need to upgrade your membership level? Please contact Customer Service at Service@asmusa.org.

The ASM Journals program strives for constant improvement in our submission and publication process. Please tell us how we can improve your experience by taking this quick Author Survey.

Thank you for submitting your paper to mSystems.

Sincerely,
Xiaoquan Su
Editor
mSystems

Reviewer #1 (Comments for the Author):

Overall, I would take this paper as a useful conceptual perspective highlighting a key technical bias in functional metagenomics, though it currently lacks strong empirical validation. The manuscript will likely be valuable for raising methodological awareness, particularly for researchers designing large-scale metagenomic studies. I generally liked the paper and believe it makes an important point. Below are several comments and suggestions that could further strengthen the manuscript.

Major comments:

1. The multiplexing argument would benefit from stronger supporting data. The central claim - that excessive multiplexing is a major cause of functional dropout - would benefit from additional supporting evidence. For example, the argument could be strengthened with more systematic benchmarking, simulated datasets and controlled sequencing experiments.
Most importantly, the authors might consider proposing or discussing a quantitative framework for estimating the relationship between sequencing coverage and multiplexing level. For instance, is there a way to estimate an optimal multiplexing ratio that accounts for factors such as total sequencing output, community diversity, and functional complexity? Even a conceptual or approximate framework for determining such thresholds would be very valuable for researchers designing sequencing experiments.

2. Cost considerations are under-discussed. Reducing multiplexing inevitably increases sequencing costs. For large population studies, the recommendations proposed in this manuscript may not always be practical. It would therefore be helpful for the authors to discuss the trade-offs between sequencing depth and cost more explicitly. In particular, discussion of potential optimal multiplexing thresholds (as mentioned above) could help researchers balance data quality with financial constraints.

3. Limited discussion of alternative solutions. The manuscript primarily focuses on sequencing design as the solution to the functional dropout problem. However, additional strategies could also help mitigate these issues and may be worth discussing briefly. For example - targeted enrichment, improved gene-calling algorithms, probabilistic pathway inference methods, assembly-based functional reconstruction approaches...etc.
Including a short discussion of these complementary strategies would provide a more balanced view of possible solutions.

Minor comments:

1. Self-citation - The manuscript contains a relatively high number of self-citations (e.g., MetaSUB, Q2PD, and the companion manuscript [31]). While these are relevant to the topic, including slightly more external examples or validation from independent studies could strengthen the argument.

2. Computational challenges section is informative but could potentially be tightened. Some readers may benefit from a brief takeaway sentence summarizing when deep learning-based methods (e.g., deepFRI) may be preferable to traditional homology-based approaches such as HUMAnN.

3. Page 2: The sentence "as a fundamental driver of healthy urban regeneration (ref)" a reference is missing here.

Reviewer #2 (Comments for the Author):

The authors provide a timely and necessary argument that suffers slightly from overstated novelty and underdeveloped economic realism. The technical recommendations are sound, but the manuscript would benefit from empirical validation within the text and a more sophisticated treatment of when high-multiplexing remains justified (e.g., pilot studies vs. definitive functional atlases).

Major comments:
1. The central premise is valid but not entirely novel.
The paper's core argument-that functional metagenomics demands higher library complexity than taxonomic profiling-is empirically sound but feels slightly overstated as a "paradigm shift." The observation that shallow sequencing misses low-abundance functions has been documented before (e.g., the Haft et al. and Thomas et al. discussions around coverage saturation). What is valuable here is framing this specifically as a multiplexing problem rather than just a "depth" problem. However, the manuscript occasionally conflates "shallow sequencing" with "high multiplexing" when these are not strictly synonymous-you can have high multiplexing with high depth per sample if you sequence multiple lanes, or low multiplexing with shallow depth if you simply sequence less. The mechanical link between pooling strategy and unique molecular coverage needs tighter experimental isolation.
2. The evidence for the "multiplexing bottleneck" is suggestive but not definitive. The authors reference an associated manuscript (Zielińska et al., bioRxiv) for the empirical demonstration that matched samples sequenced with different protocols show functional dropout at high multiplexing. Without those data presented here, this Perspective relies heavily on hypothetical saturation curves (Figure 1C) and general principles of PCR duplication. While the logic is biologically plausible, the paper would be stronger with even a minimal illustrative dataset showing, for example, the same stool sample sequenced at 8-plex versus 96-plex with comparable total reads, demonstrating the loss of unique gene detection. As it stands, the argument rests on extrapolation from RNA-seq literature (Zajac et al.) rather than metagenomics-specific empirical proof.
3. The cost-benefit analysis is hand-waved. The recommendation to "deliberately reduce multiplexing levels" is, practically speaking, a recommendation to increase costs by an order of magnitude. For large-scale population studies (like the MetaSUB

consortium mentioned), dropping from 384-plex to 48-plex means 8x more sequencing lanes, which transforms a $50,000 project into a $400,000 one. The authors acknowledge this only obliquely ("cost-effective compromise") without seriously grappling with the reality that funding agencies often prioritize sample size (n=10,000) over molecular resolution. A more nuanced discussion of information-per-dollar rather than just information-per-sample would strengthen the pragmatic value of this perspective.

4. The taxonomic-vs-functional robustness claim oversimplifies. The assertion that "taxonomic assignments can be relatively resilient to reduced depths" while functional profiling suffers dropouts is generally true for coarse taxonomy (phylum/genus level), but falls apart at the strain-level resolution the authors rightly prioritize. Modern metagenomic strain profiling (via inStrain, StrainPhlAn, or similar) is extremely sensitive to coverage and suffers similar stochastic dropouts as functional genes. The dichotomy between "robust taxonomy" and "fragile function" creates a false sense of security-if you're doing serious strain-resolved metagenomics (which you need for AMR tracking), taxonomy is just as brittle as function at low coverage.

5. Long-read evangelism understates the bioinformatics burden. The push for long-read or hybrid architectures is welcome, but the paper glosses over the current state of functional annotation from long-read metagenomes. While long reads span operons beautifully, the per-base error rates (even with Q20+ chemistry) still create headaches for precise functional assignment at the nucleotide level, particularly for AMR gene variant identification where single SNPs matter. The "hybrid" approach is sensible but computationally expensive-requiring both short-read polishing and long-read assembly infrastructure that many labs lack. A brief acknowledgment that long-read functional metagenomics is still a specialist technique, not a turnkey solution, would balance this section.

6. The exposome/One Health framing feels grafted on. The section connecting multiplexing ratios to exposome monitoring and "microbial digital twins" is conceptually exciting but logically tenuous. The leap from library preparation technicalities to global health surveillance happens too quickly, without establishing why slightly higher duplication rates in a sequencing library would materially compromise epidemiological conclusions at the population level. If the argument is that we miss rare AMR genes, that's valid, but the connection to "regenerative urban design" (MUSA project) feels like project promotion rather than organic intellectual development.

7. Missing: the "reference bias" angle. While the authors correctly note that homology-based functional annotation is limited by database completeness, they don't fully connect this to their multiplexing argument. High multiplexing doesn't just cause dropouts-it systematically biases against rare, lineage-specific genes that are already underrepresented in reference databases. This creates a compounding effect: technical dropout meets annotation darkness. Explicitly stating that low-complexity libraries reinforce reference database biases would strengthen the urgency of their recommendations.

8. Figure 1: pedagogically helpful but schematically imprecise

Panel B implies that high multiplexing causes "competitive binding" during library prep, which is slightly misleading. The issue isn't competitive binding in the hybridization sense, but rather sampling noise during template distribution and PCR bottleneck effects. The visual metaphor of "fewer molecules per sample" is accurate, but the mechanism should be clarified-it's about stochastic sampling during emPCR/bridge amplification, not competitive inhibition.

9. The Global South point is performative. The mention of under-sampling in the Global South (page 4) appears abruptly and disappears just as quickly. If the authors want to make a point about equity in microbiome science, it needs either elaboration-connecting how high-multiplexing, low-depth strategies disproportionately affect studies in underrepresented populations where novel functions are most likely to be found-or removal as a distracting aside.

10. Strengths worth preserving. Despite these critiques, the paper serves an important corrective function. The field has indeed been "multiplexing drunk" since 2015, chasing sample size at the expense of biological signal. The emphasis on unique coverage rather than raw read counts is a crucial methodological reminder. The recommendations-particularly the hybrid sequencing approach and the caution against rarefaction as a normalization crutch-are pragmatic and implementable. If the authors tightened the empirical backing and confronted the cost realities more honestly, this could become a definitive methodological benchmark.

Reviewer #1 (Comments for the Author):

Overall, I would take this paper as a useful conceptual perspective highlighting a key technical bias in functional metagenomics, though it currently lacks strong empirical validation. The manuscript will likely be valuable for raising methodological awareness, particularly for researchers designing large-scale metagenomic studies. I generally liked the paper and believe it makes an important point. Below are several comments and suggestions that could further strengthen the manuscript.

Major comments:

1. The multiplexing argument would benefit from stronger supporting data. The central claim - that excessive multiplexing is a major cause of functional dropout - would benefit from additional supporting evidence. For example, the argument could be strengthened with more systematic benchmarking, simulated datasets and controlled sequencing experiments.
Most importantly, the authors might consider proposing or discussing a quantitative framework for estimating the relationship between sequencing coverage and multiplexing level. For instance, is there a way to estimate an optimal multiplexing ratio that accounts for factors such as total sequencing output, community diversity, and functional complexity? Even a conceptual or approximate framework for determining such thresholds would be very valuable for researchers designing sequencing experiments.

We thank the Reviewer for this thoughtful comment. We would like to clarify that the empirical evidence supporting our central claim is provided in detail in the companion manuscript [32] (Zielińska et al., now accepted), which was submitted alongside this Perspective and is referenced throughout. The Perspective was intentionally written as a concise conceptual piece that builds on those findings, rather than as a standalone empirical report.
The companion study [32] provides precisely the kind of systematic benchmarking the Reviewer suggests. It is not a small-scale pilot - it comprises 1,351 matched human gut metagenomic sample pairs from the same individuals, sequenced on both the Illumina NovaSeq 6000 (high multiplexing, ~30M reads/sample) and MGISEQ-2000 (low multiplexing, ~108M reads/sample) platforms. The key findings that directly support the multiplexing argument in this Perspective include:

- **Unique molecular complexity is dramatically reduced under high multiplexing, independent of read depth.** After rarefaction to identical read counts, MGI samples (prepared with lower multiplexing) retained 5–10× more unique k-mers than their NovaSeq counterparts (Figure 5d in [32]). This demonstrates that the issue is library complexity - a direct consequence of multiplexing level - not sequencing depth per se.
- **Functional profiles are disproportionately affected while taxonomy remains stable.** Functional similarity within matched pairs ranged from 80–97% (Figure 5c in [32]), compared to >92% taxonomic overlap. Critically, there was no correlation between taxonomic and functional overlap (Spearman $\rho = 0.005$, $p > 0.05$; Figures 5c and 5g in [32]), confirming that functional dropout is decoupled from taxonomic variation and instead reflects library-level artifacts.
- **The functional bias is systematic and pervasive.** Differential abundance analysis identified 241 enzymes significantly enriched in one platform (FDR-adjusted $p < 0.05$), with 78% of the 100 most differentially abundant enzymes showing higher abundance in Illumina samples (Figure 5a, 5b in [32]). These biases affected core metabolic pathways - carbohydrate metabolism, amino acid biosynthesis, and energy/cofactor metabolism - not just rare or low-prevalence functions.

- **Rarefaction does not resolve the problem.** Simulated rarefaction analysis (Figure 5h in [32]) showed that inter-platform functional distances persisted even after normalizing to equal read depth, remaining approximately double the distance between technical replicates. This confirms that the standard computational correction (rarefaction) is insufficient to mitigate library complexity–driven biases.
- **Direct visualization of coverage gaps.** Genome coverage analysis of representative gut species revealed that regions well-covered by one platform were entirely missing in the other (Supplementary Figure 5 in [32]), providing a mechanistic explanation for the observed functional dropout.

Regarding the Reviewer's suggestion to propose a quantitative framework for estimating optimal multiplexing ratios as a function of total sequencing output, community diversity, and functional complexity - we agree this is an important and valuable direction. However, deriving such a framework would require systematic titration experiments across multiple community types and sequencing configurations, which we believe constitutes a full research programme in its own right, beyond the scope of this Perspective. That said, we recognize the value of acknowledging this explicitly. We have added a brief statement in the Recommendations section (lines 212–221) noting that our empirical findings in [32] provide a foundation for developing such a quantitative framework, and that this represents a key next step for the field.

2. Cost considerations are under-discussed. Reducing multiplexing inevitably increases sequencing costs. For large population studies, the recommendations proposed in this manuscript may not always be practical. It would therefore be helpful for the authors to discuss the trade-offs between sequencing depth and cost more explicitly. In particular, discussion of potential optimal multiplexing thresholds (as mentioned above) could help researchers balance data quality with financial constraints.

We appreciate the Reviewer raising this important practical consideration. We agree that cost is a key factor in experimental design and that our recommendations should be contextualized within realistic financial constraints.

However, we would like to reframe the discussion slightly. Our central argument is not that researchers should simply sequence fewer samples - it is that the field needs to be aware that high multiplexing comes at a hidden cost: the systematic loss of functional information. This is a cost that is currently invisible to most researchers, because taxonomic profiles appear unaffected. In practice, many studies that have invested substantially in sequencing large cohorts may have inadvertently generated data from which reliable functional inference is not possible. This represents a significant, if unrecognized, waste of resources in itself.

Furthermore, the cost landscape is shifting rapidly. Sequencing costs per gigabase continue to decline, and newer platforms increasingly offer higher output per run. What was prohibitively expensive five years ago is becoming routine. Our recommendation to reduce multiplexing is therefore not a call to spend more indefinitely, but rather to allocate sequencing resources more thoughtfully - prioritizing per-sample library complexity over sheer sample number where functional analysis is the goal. For studies where only taxonomic profiling is intended, high multiplexing remains entirely appropriate, as our own data in [32] demonstrate (>92% species overlap between platforms despite vastly different multiplexing levels; Table 1 and Figure 4 in [32]).

We also note that the hybrid sequencing approach we already recommend in the Perspective (combining deep short-read with targeted long-read data) is itself a cost-conscious strategy - it explicitly aims to improve functional resolution without requiring that every sample be sequenced at maximum depth on a single platform.

That said, we recognize the value of making these trade-offs more explicit in the text. We have added a brief acknowledgment in the Recommendations section (lines 222–234) noting that the optimal balance between multiplexing level and functional recovery will depend on the specific aims of the study, and that for projects where functional profiling is not the primary

objective, higher multiplexing ratios remain a valid and cost-effective strategy. This pairs naturally with the text on quantitative frameworks we proposed to add in response to Comment 1 - together, they frame multiplexing as a design variable to be optimized rather than universally minimized.

3. Limited discussion of alternative solutions. The manuscript primarily focuses on sequencing design as the solution to the functional dropout problem. However, additional strategies could also help mitigate these issues and may be worth discussing briefly. For example - targeted enrichment, improved gene-calling algorithms, probabilistic pathway inference methods, assembly-based functional reconstruction approaches...etc.
Including a short discussion of these complementary strategies would provide a more balanced view of possible solutions.

We thank the Reviewer for this suggestion and agree the Perspective would benefit from a broader acknowledgment of complementary strategies. We note that the "Computational challenges" section already discusses several emerging alternatives to homology-based annotation, including deep learning–based methods (deepFRI), enzyme-specific tools (PARSE, CLEAN), and structure-based function prediction via AlphaFold - presented precisely as complementary approaches to overcome current annotation limitations.

We have expanded the Recommendations section (lines 265-269) to additionally acknowledge wet-lab complementary strategies: specifically, that targeted enrichment of functional gene classes (e.g., AMR gene panels) can provide focused resolution where comprehensive whole-metagenome coverage is not achievable, and that assembly-based approaches, while valuable, remain dependent on the same underlying library complexity and do not eliminate the need for adequate unique coverage.

Minor comments:

1. Self-citation - The manuscript contains a relatively high number of self-citations (e.g., MetaSUB, Q2PD, and the companion manuscript [32]). While these are relevant to the topic, including slightly more external examples or validation from independent studies could strengthen the argument.

We thank the Reviewer for this observation. We would like to briefly address each self-citation cluster separately, as they arise for different reasons.
The companion manuscript [32] represents our own primary empirical dataset and cannot be substituted by external citations - it is cited because it is the direct evidence for the claims being made. Similarly, references [11, 13–15] reflect our own contributions to the MetaSUB consortium and are cited in the context of describing work we participated in. We have reduced this cluster slightly and added an external example of WMS-based AMR surveillance (line 44, reference [12]) to broaden the supporting evidence base.

For reference [16] (Q2PD), where we argue that functional potential is more conserved across global populations than taxonomic composition, we agree that independent external support strengthens the claim. We have therefore added Turnbaugh et al. (2009, Nature) (lines 59–63), which showed in a landmark twin study that functional gene categories are substantially more conserved than species-level composition - providing an independent, widely-cited line of evidence for this argument.

Finally, we note that our analogy to RNA-seq library complexity (Zajac et al. [28]) was cited as the closest methodological parallel available at the time of writing. We have now added Hillmann et al. (2018, mSystems) as a metagenomics-specific complement (lines 107–111), which demonstrated that shallow shotgun sequencing imposes a fundamental ceiling on functional analysis not shared by taxonomic profiling - specifically the inability to assemble novel genes and genomic regions de novo, a constraint that does not apply equally to marker-based taxonomic approaches.

2. Computational challenges section is informative but could potentially be tightened. Some readers may benefit from a brief takeaway sentence summarizing when deep learning-based methods (e.g., deepFRI) may be preferable to traditional homology-based approaches such as HUMAnN.

We thank the Reviewer for this suggestion. We have tightened the section and added a brief takeaway sentence (lines 186–189) clarifying when deep learning-based approaches offer advantages over homology-based methods.

3. Page 2: The sentence "as a fundamental driver of healthy urban regeneration (ref)" a reference is missing here.

Thank you for this observation – we have now added the reference.

Reviewer #2 (Comments for the Author):

The authors provide a timely and necessary argument that suffers slightly from overstated novelty and underdeveloped economic realism. The technical recommendations are sound, but the manuscript would benefit from empirical validation within the text and a more sophisticated treatment of when high-multiplexing remains justified (e.g., pilot studies vs. definitive functional atlases).

Major comments:

1. The central premise is valid but not entirely novel.

The paper's core argument-that functional metagenomics demands higher library complexity than taxonomic profiling-is empirically sound but feels slightly overstated as a "paradigm shift." The observation that shallow sequencing misses low-abundance functions has been documented before (e.g., the Haft et al. and Thomas et al. discussions around coverage saturation). What is valuable here is framing this specifically as a multiplexing problem rather than just a "depth" problem. However, the manuscript occasionally conflates "shallow

sequencing" with "high multiplexing" when these are not strictly synonymous-you can have high multiplexing with high depth per sample if you sequence multiple lanes, or low multiplexing with shallow depth if you simply sequence less. The mechanical link between pooling strategy and unique molecular coverage needs tighter experimental isolation.

We thank the Reviewer for this precise and constructive critique. We agree on all three points and respond to each in turn.

First, we accept that "paradigm shift" overstates the novelty of the observation that shallow sequencing affects functional recovery. We have softened this language. What we maintain as a meaningful and actionable contribution is the mechanistic reframing: the root cause is not sequencing depth per se, but library complexity - the number of unique molecules captured during library preparation - which is directly governed by multiplexing strategy. This distinction has practical consequences that a depth-centred framing does not capture.

Second, we agree that the perspective at times conflates shallow sequencing with high multiplexing, and we revised the relevant passages to sharpen the causal chain (lines 112–139). The argument we intend to make is: high multiplexing reduces per-sample input → library complexity is reduced → duplication rates rise → coverage becomes uneven → functional genes are stochastically missed. Critically, a library with compromised complexity cannot be rescued computationally, because additional reads from the same pool will disproportionately re-sequence already-represented molecules rather than recovering missing ones. This means the problem is upstream and irreversible, not merely a matter of insufficient read count.

Third, regarding the request for tighter experimental isolation of the multiplexing effect: we would direct the Reviewer to Figure 5d and 5h of the companion manuscript [32], which provide precisely this. After rarefaction to identical read depths, MGI samples - prepared with lower multiplexing - retained 5–10× more unique k-mers than their NovaSeq counterparts, and inter-platform functional distances persisted despite matched depth. This demonstrates that library complexity, not depth, is the operative variable. We made this mechanistic distinction more explicit in the perspective text itself (lines 128–139).

2. The evidence for the "multiplexing bottleneck" is suggestive but not definitive. The authors reference an associated manuscript (Zielińska et al., bioRxiv) for the empirical demonstration that matched samples sequenced with different protocols show functional dropout at high multiplexing. Without those data presented here, this Perspective relies heavily on hypothetical saturation curves (Figure 1C) and general principles of PCR duplication. While the logic is biologically plausible, the paper would be stronger with even a minimal illustrative dataset showing, for example, the same stool sample sequenced at 8-plex versus 96-plex with comparable total reads, demonstrating the loss of unique gene detection. As it stands, the argument rests on extrapolation from RNA-seq literature (Zajac et al.) rather than metagenomics-specific empirical proof.

We thank the Reviewer for this comment. We would like to clarify that the companion manuscript [32] is not a peripheral supporting reference - it is the direct empirical demonstration of the claim. It comprises 1,351 matched human gut metagenomic sample pairs from the same individuals, sequenced on two platforms with substantially different multiplexing levels: Illumina NovaSeq 6000 (high multiplexing, ~30M reads/sample) and MGISEQ-2000 (low multiplexing, ~108M reads/sample). This is precisely the kind of controlled matched-sample comparison the Reviewer describes, applied at population scale

rather than in a single-sample titration. The manuscript [32] is accepted for publication at mSystems and we intend to release both back-to-back precisely as supporting evidence.
Specifically, the data in [32] address the Reviewer's request in the following ways:
The k-mer analysis (Figure 5d in [32]) shows that after rarefaction to identical read depths, MGI samples retained 5–10× more unique k-mers than their NovaSeq counterparts. This is the metagenomics-specific empirical demonstration that library complexity - not read depth - is the operative variable, and that it is directly governed by multiplexing strategy.
The rarefaction simulation (Figure 5h in [32]) shows that inter-platform functional distances persisted at approximately double the intra-platform distance even after matching read depth, confirming that the functional discrepancy cannot be attributed to differences in sequencing depth and instead reflects upstream library complexity differences.
The genome coverage analysis (Supplementary Figure 5 in [32]) shows non-overlapping coverage patterns between the two platforms at identical read depths, with entire genomic regions present in one protocol entirely absent in the other - a direct visualization of the stochastic dropout mechanism.
We acknowledge, however, that the Reviewer's observation has a legitimate practical implication: the Perspective text itself does not present any of these numbers, meaning readers without immediate access to [32] encounter only schematic figures and an RNA-seq analogy. We have addressed this by adding a brief passage in the “Challenges” section (lines 133–139) that summarizes the headline quantitative findings from [32] directly, making the empirical grounding visible within the perspective itself.
Regarding Figure 1C specifically: we accept that the saturation curves as currently presented are schematic and conceptual. We have revised the figure legend (lines 292-297) to make clear that these are illustrative representations of the relationships demonstrated empirically in [32], rather than data-derived curves, and added a direct reference to the relevant figures in [32] for readers seeking the underlying data.
We would also note that the Reviewer's suggestion of an "8-plex versus 96-plex" titration experiment, while methodologically elegant, would itself constitute a full research programme beyond the scope of this manuscript. The matched-sample design in [32] - where the same biological material was processed through two different multiplexing protocols and compared at identical read depths - provides the controlled isolation of the multiplexing effect that the Reviewer is asking for.

3. The cost-benefit analysis is hand-waved. The recommendation to "deliberately reduce multiplexing levels" is, practically speaking, a recommendation to increase costs by an order of magnitude. For large-scale population studies (like the MetaSUB consortium mentioned), dropping from 384-plex to 48-plex means 8x more sequencing lanes, which transforms a $50,000 project into a $400,000 one. The authors acknowledge this only obliquely ("cost-effective compromise") without seriously grappling with the reality that funding agencies often prioritize sample size (n=10,000) over molecular resolution. A more nuanced discussion of information-per-dollar rather than just information-per-sample would strengthen the pragmatic value of this perspective.

We thank the Reviewer for this detailed and concrete engagement with the cost question. The specific numbers raised - 384-plex to 48-plex representing an 8× cost increase - are real and we do not dispute them. We agree that our original text handled this too briefly, and we address it more substantively in the revised manuscript.

However, we would like to reframe the economics, because we believe the "information-per-dollar" lens the Reviewer correctly invokes actually strengthens our argument rather than

undermining it. The relevant question is not the cost per sample sequenced, but the cost per valid functional data point generated. A $50,000 study conducted at 384-plex may produce taxonomic data of high quality, but if functional profiles generated from the same run are systematically biased - with 241 enzymes showing significant platform-dependent enrichment and functional overlaps between matched samples as low as 80% (as demonstrated in [32]) - then the functional information content of that $50,000 investment is substantially compromised. The money has been spent; the functional data cannot be trusted. From an information-per-dollar perspective, this is a poor outcome regardless of sample size.

We would also push back gently on the framing of this as an either/or choice. The Reviewer presents it as 384-plex (affordable, n=10,000) versus 48-plex (unaffordable, n=10,000). But a more practical interpretation of our recommendation is a tiered design: use high multiplexing for the full cohort where taxonomic profiling is the goal, and reserve lower-multiplexing deep sequencing for a well-powered functional subset. Many large-scale studies already implicitly adopt this logic through pilot sequencing or targeted deep-dives. Making this strategy explicit and principled - informed by power calculations for functional gene detection rather than taxonomic diversity - is precisely the kind of guidance we are advocating for.

Furthermore, the specific cost calculus is changing rapidly. Per-gigabase sequencing costs have fallen by several orders of magnitude over the past decade and continue to decline. Platforms offer dramatically higher output per run, meaning that achieving deeper per-sample coverage at lower multiplexing is becoming increasingly accessible. A cost argument that was prohibitive in 2018 may be merely inconvenient in 2026 and routine by 2028.

We have revised the Recommendations section (lines 226-238) to acknowledge these trade-offs explicitly, including the tiered design strategy and the information-per-dollar framing, making the practical guidance more actionable for researchers operating under real funding constraints.

4. The taxonomic-vs-functional robustness claim oversimplifies. The assertion that "taxonomic assignments can be relatively resilient to reduced depths" while functional profiling suffers dropouts is generally true for coarse taxonomy (phylum/genus level), but falls apart at the strain-level resolution the authors rightly prioritize. Modern metagenomic strain profiling (via inStrain, StrainPhlAn, or similar) is extremely sensitive to coverage and suffers similar stochastic dropouts as functional genes. The dichotomy between "robust taxonomy" and "fragile function" creates a false sense of security-if you're doing serious strain-resolved metagenomics (which you need for AMR tracking), taxonomy is just as brittle as function at low coverage.

We thank the Reviewer for this precise and important clarification. We agree that the taxonomy-versus-function robustness dichotomy as stated is an oversimplification, and we have revised the text accordingly (lines 121–127). The resilience of taxonomic profiling to reduced coverage applies at coarse taxonomic levels - species, genus, and above - where assignments are supported by multiple genomic regions and redundant marker sequences. At the strain level, as the Reviewer correctly notes, this resilience disappears entirely. Modern strain profiling tools such as inStrain and StrainPhlAn require substantial, uniform per-genome coverage to call variants reliably, and are therefore just as sensitive to library complexity loss as functional gene detection.

We would note, however, that this observation reinforces rather than undermines our core recommendations. If strain-level taxonomic resolution - which the Perspective explicitly prioritizes for applications such as AMR tracking - suffers the same stochastic dropouts as functional profiling under high multiplexing, then both goals converge on the same solution: reducing multiplexing to recover library complexity. The Reviewer's point effectively extends the scope of the problem we are describing, making the case for our recommendations broader and more urgent.

We have revised the relevant passage (lines 121–127) to specify that taxonomic resilience applies at the species and genus level, and to explicitly acknowledge that strain-level profiling is subject to the same coverage constraints as functional annotation - further motivating the experimental design changes we propose.

5. Long-read evangelism understates the bioinformatics burden. The push for long-read or hybrid architectures is welcome, but the paper glosses over the current state of functional annotation from long-read metagenomes. While long reads span operons beautifully, the per-base error rates (even with Q20+ chemistry) still create headaches for precise functional assignment at the nucleotide level, particularly for AMR gene variant identification where single SNPs matter. The "hybrid" approach is sensible but computationally expensive-requiring both short-read polishing and long-read assembly infrastructure that many labs lack. A brief acknowledgment that long-read functional metagenomics is still a specialist technique, not a turnkey solution, would balance this section.

We thank the Reviewer for this grounded and important correction. We agree that the long-read section as written is somewhat uncritical and glosses over real and current limitations. We have added a brief acknowledgment of these constraints (lines 245–248) to balance the recommendation.

Specifically, we accept the following points: first, that per-base error rates in long-read sequencing - even with Q20+ chemistry - remain a practical concern for precise functional assignment at the nucleotide level, and are particularly problematic for AMR gene variant identification where single SNPs determine resistance profiles and misclassification has direct clinical implications. Second, the hybrid approach, while conceptually sound, involves both short-read polishing infrastructure and long-read assembly pipelines that require significant amounts of computational resources and time. However, we would note that the long-read sequencing landscape is evolving rapidly, and we believe the Reviewer's characterisation, while accurate for the recent past, may already be partially overtaken by developments in the field. Per-base error rates have improved dramatically with recent chemistry advances, bringing them close to those of short-read technologies, and - notably - some long-read sequencing platforms have recently received approval for diagnostic use, suggesting that the accuracy barrier is being addressed at a pace that may surprise. We therefore retain the view that long-read functional metagenomics, while not yet a turnkey solution, is moving toward becoming one, and that our recommendations are best understood as directional guidance for the field rather than immediately actionable prescriptions for every research group. We have revised the text (lines 245–264) to reflect this balanced position - acknowledging the current practical limitations while noting that continued improvements in long-read accuracy and the development of dedicated metagenomic long-read pipelines will progressively lower these barriers.

6. The exposome/One Health framing feels grafted on. The section connecting multiplexing ratios to exposome monitoring and "microbial digital twins" is conceptually exciting but

logically tenuous. The leap from library preparation technicalities to global health surveillance happens too quickly, without establishing why slightly higher duplication rates in a sequencing library would materially compromise epidemiological conclusions at the population level. If the argument is that we miss rare AMR genes, that's valid, but the connection to "regenerative urban design" (MUSA project) feels like project promotion rather than organic intellectual development.

We thank the Reviewer for this frank assessment.
On the logical connection: we agree that the current text moves too quickly from library preparation technicalities to population-level consequences without establishing the causal chain explicitly. The argument we intend to make - but have not adequately articulated - is the following: the functional genes most vulnerable to stochastic dropout under high multiplexing are, by definition, those present at low abundance in the community. This category disproportionately includes horizontally transferred elements, lineage-specific AMR determinants, and genes associated with emerging or rare resistance mechanisms - precisely the signals of greatest epidemiological importance. Missing these genes systematically and reproducibly across large cohorts does not merely introduce noise; it creates blind spots in AMR surveillance that could delay detection of emerging resistance hotspots or mask cross-environment transfer of resistance determinants. We have restructured this section (lines 74–87) to make this causal chain explicit before invoking the broader exposome and digital twins framing. We agree that the section about the MUSA project did not fit the neighbouring parts - we have removed this fragment.

7. Missing: the "reference bias" angle. While the authors correctly note that homology-based functional annotation is limited by database completeness, they don't fully connect this to their multiplexing argument. High multiplexing doesn't just cause dropouts-it systematically biases against rare, lineage-specific genes that are already underrepresented in reference databases. This creates a compounding effect: technical dropout meets annotation darkness. Explicitly stating that low-complexity libraries reinforce reference database biases would strengthen the urgency of their recommendations.

We thank the Reviewer for this observation, which we find genuinely strengthening of our argument. The reviewer has identified a logical connection between the two main threads of the Perspective - the experimental and the computational - that we had not made explicit. We agree entirely and have added this explicitly to the text (lines 167–178).
The compounding effect the Reviewer describes operates as follows: high multiplexing causes stochastic dropout that disproportionately affects rare, low-abundance, and lineage-specific genes. These are, by definition, the same genes that are most poorly represented or entirely absent from existing reference databases - since reference databases are themselves built from the genes most frequently detected and annotated across studies, which are the abundant ones. A library with compromised complexity therefore simultaneously reduces the probability of capturing these rare genes in sequencing and, if they are captured at reduced coverage, reduces the probability of annotating them correctly against an incomplete reference. The result is a systematic and self-reinforcing bias: the most technically challenging genes to detect are also the most computationally challenging to annotate, and high multiplexing amplifies both problems at once. Crucially, this compounding effect means that computational advances - improved alignment algorithms, expanded databases, deep learning–based annotation - cannot fully compensate for the upstream library complexity problem, because you cannot annotate

molecules that were never sequenced. This further reinforces the urgency of our wet-lab recommendations.

8. Figure 1: pedagogically helpful but schematically imprecise
Panel B implies that high multiplexing causes "competitive binding" during library prep, which is slightly misleading. The issue isn't competitive binding in the hybridization sense, but rather sampling noise during template distribution and PCR bottleneck effects. The visual metaphor of "fewer molecules per sample" is accurate, but the mechanism should be clarified-it's about stochastic sampling during emPCR/bridge amplification, not competitive inhibition.

We thank the Reviewer for this precise mechanistic correction. We agree that the phrase "competitive binding" in the Figure 1B legend is misleading and implies a hybridization-based competition that does not occur in standard shotgun library preparation. We have revised the legend (lines 286-292) to accurately describe the mechanism: high multiplexing reduces per-sample input material, which means rare molecules are frequently absent from the starting pool by stochastic chance - sequences present at low copy number in the input will often simply not be sampled. PCR amplification then over-represents whatever molecules were captured, producing high duplication rates and a library that appears to have complexity it does not. The result is probabilistic absence of rare sequences from the library, not competitive exclusion. The visual metaphor of "fewer molecules per sample" in Panel B is retained as it correctly represents the outcome, but we will clarify the underlying mechanism in the legend.

8. The Global South point is performative. The mention of under-sampling in the Global South (page 4) appears abruptly and disappears just as quickly. If the authors want to make a point about equity in microbiome science, it needs either elaboration-connecting how high-multiplexing, low-depth strategies disproportionately affect studies in underrepresented populations where novel functions are most likely to be found-or removal as a distracting aside.

Fair point. We have removed the Global South reference.

10. Strengths worth preserving. Despite these critiques, the paper serves an important corrective function. The field has indeed been "multiplexing drunk" since 2015, chasing sample size at the expense of biological signal. The emphasis on unique coverage rather than raw read counts is a crucial methodological reminder. The recommendations-particularly the hybrid sequencing approach and the caution against rarefaction as a normalization crutch-are pragmatic and implementable. If the authors tightened the empirical backing and confronted the cost realities more honestly, this could become a definitive methodological benchmark.

We thank Reviewer for these encouraging remarks and for the constructive framing of their critique throughout. We are glad that the core argument - that unique molecular coverage rather than raw read depth is the operative variable for functional metagenomics - is recognized as a valuable methodological contribution. In the revised manuscript we have directly addressed both of the conditions the Reviewer identifies: the empirical backing has been tightened by integrating the headline quantitative findings from [32] into the Perspective text itself (lines 133–139), and the cost trade-offs are now discussed more explicitly and with greater nuance, including the information-per-dollar framing and the tiered study design

strategy (lines 226–238). We hope the revised Perspective meets the standard the Reviewer envisions.

Re: mSystems00144-26R1 (Moving from taxonomic to functional perspective in global microbiome analysis requires optimizing multiplexing ratios)

Dear Dr. Tomasz Kosciolek:

Your manuscript has been accepted, and I am forwarding it to the ASM production staff for publication. Your paper will first be checked to make sure all elements meet the technical requirements. ASM staff will contact you if anything needs to be revised before copyediting and production can begin. Otherwise, you will be notified when your proofs are ready to be viewed.

Data Availability: ASM policy requires that data be available to the public upon online posting of the article, so please verify all links to sequence records, if present, and make sure that each number retrieves the full record of the data. If a new accession number is not linked or a link is broken, provide production staff with the correct URL for the record. If the accession numbers for new data are not publicly accessible before the expected online posting of the article, publication may be delayed; please contact ASM production staff immediately with the expected release date.

Publication Fees: For information on publication fees and which article types have charges, please visit our website. We have partnered with Copyright Clearance Center (CCC) to collect author charges. If fees apply to your paper, you will receive a message from no-reply@copyright.com with further instructions. For questions related to paying charges through RightsLink, please contact CCC at ASM_Support@copyright.com or toll free at +1-877-622-5543. CCC makes every attempt to respond to all emails within 24 hours.

ASM Membership: Corresponding authors may join or renew ASM membership to obtain discounts on publication fees. Need to upgrade your membership level? Please contact Customer Service at Service@asmusa.org.

Embargo Policy: A press release may be issued as soon as the manuscript is posted on the mSystems Latest Articles webpage. The corresponding author will receive an email with the subject line "ASM Journals Author Services Notification" when the article is available online.

Cover Image Submissions: If you would like to submit a potential Cover Image, please email a file and a short legend to msystems@asmusa.org. Please note that we can only consider images that (i) the authors created or own and (ii) have not been previously published. By submitting, you agree that the image can be used under the same terms as the published article. Image File requirements: TIF/EPS, 7.5 inches wide by 8.25 inches tall (at least 2,250 pixels wide by 2,475 pixels tall), minimum 300 dpi resolution (600 dpi preferred), RGB, and no figure elements, e.g., arrows or panel labels. The legend should be a short description of the image, 1-2 sentences recommended. Please download and use this interactive template in Adobe to ensure that your proposed cover image meets our size requirements (https://journals.asm.org/pb-assets/pdf-text-excel-files/ASM-Interactive-Sizing-Cover-Template-1715689791.pdf).

Thank you for submitting your paper to mSystems.

Sincerely,
Xiaoquan Su
Editor
mSystems

Reviewer #1 (Comments for the Author):

I appreciate that the authors have addressed all my comments properly. I don't have further questions

Reviewer #2 (Comments for the Author):

No further comments from me.
